# Asymptomatic Primary Merkel Cell Polyomavirus Infection among Adults

**DOI:** 10.3201/eid1708.110079

**Published:** 2011-08

**Authors:** Yanis L. Tolstov, Alycia Knauer, Jian Guo Chen, Thomas W. Kensler, Lawrence A. Kingsley, Patrick S. Moore, Yuan Chang

**Affiliations:** Author affiliations: University of Pittsburgh, Pittsburgh, Pennsylvania, USA (Y.L. Tolstov, A. Knauer, T.W. Kensler, L.A. Kingsley, P.S. Moore, Y. Chang);; Qidong Liver Cancer Institute, Jiangsu, People’s Republic of China (J.G. Chen);; Johns Hopkins University, Baltimore, Maryland, USA (T.W. Kensler)

**Keywords:** viruses, MSM, men who have sex with men, Merkel cell polyomavirus, MCV, Merkel cell carcinoma, MCPyV, epidemiology, seroconversion, Pennsylvania, USA, China, HIV/AIDS and other retroviruses, research

## Abstract

Merkel cell polyomavirus (MCV) is a recently discovered virus that causes 80% of Merkel cell carcinomas. We examined data for 564 gay/bisexual male participants >18 years of age in the Multicenter AIDS Cohort Study in Pittsburgh, Pennsylvania, USA, and found that 447 (79.3%) were MCV-antibody positive at initial enrollment. Of the 117 MCV-seronegative men, 31 subsequently seroconverted over a 4-year follow-up period, corresponding to a 6.6% annual conversion rate. MCV immunoglobulin G levels remained detectable up to 25 years after exposure. No signs, symptoms, or routine diagnostic test results were associated with MCV infection, and no correlation between HIV infection or AIDS progression and MCV infection was noted. An initial correlation between chronic hepatitis B virus infection and MCV prevalence could not be confirmed among MCV seroconverters or in studies of a second hepatitis B virus–hyperendemic cohort from Qidong, China. In adults, MCV is typically an asymptomatic, common, and commensal viral infection that initiates rare cancers after virus (rather than host cell) mutations.

## Abstract 2

TOC Summary: This virus may be part of normal human flora and harmless in most adults.

Merkel cell carcinoma (MCC) is a rare but aggressive skin cancer most commonly occurring among the elderly and among immunosuppressed persons, including AIDS patients (*1**–**3*). By using digital transcriptome subtraction, Feng et al. recently discovered Merkel cell polyomavirus (MCV) clonally integrated in the tumor cell genome of ≈80% of MCC (*4*). This association between MCV and MCC has subsequently been confirmed by other investigators (*5**–**8*). MCV in MCC tumors possesses specific mutations that disable virus replication (*9*), which indicates that MCV is not a passenger virus and provides an explanation for how a common infection can lead to a rare tumor. MCV T antigen is specifically expressed in MCV-positive MCC tumor cells (*10*). T-antigen knockdown studies show that MCV T antigen is needed for the tumor phenotype in MCV-positive tumor cells (*11*), and the extent of tumors in the patient is correlated with levels of antibodies to MCV T antigen (*12*), leaving little doubt that MCV is the infectious cause for most but not all MCC tumors.

Serologic studies have been the primary tool to investigate the prevalence of various polyomaviruses in human populations (*13**–**16*). BK virus (BKV) and John Cunningham virus (JCV), for example, are ubiquitous human polyomavirus infections. Seroconversion for both occurs largely in childhood, with BKV seroprevalence reaching 75% among children >9 years of age and JCV seroprevalence estimated at >23% among those >10 years of age (*17*). The seroprevalence of Washington University and Karolinska Institute polyomaviruses plateau at 56% and 54%, respectively, for children 5–9 years of age (*17*). Longitudinal studies measuring immunoglobulin (Ig) G to BKV show stable levels throughout life with a slight tendency to decline after age 40–50 years, while JCV seropositivity tends to increase slowly from childhood into late adulthood (*13**,**14**,**17**,**18*). A serologic study of adult commercial blood donors that used human polyomavirus 6 and 7 virus-like particles showed that these viruses are also widely established in the human population with 69% and 35% seroprevalence, respectively (*19*).

Despite numerous reports describing seroprevalence for human polyomaviruses, less is known about seroconversion or signs and symptoms of primary polyomavirus infection (*13**,**14**,**20*). Two studies have reported JCV and BKV seroconversion among adults (*13**,**20*), but no data were presented describing signs or symptoms associated with infection. Bohl et al. demonstrated that antibody titers among kidney transplant donors reflect the activity and transmissibility of BKV infection (*21**,**22*). Randhawa et al. reported an inverse correlation between serum anti-BKV IgG optical density (OD) and peak urine viral load in kidney transplant recipients, suggesting a possible protective role of serum antibodies, which may impact the clinical outcome of postransplant BKV infection (*20*). These reports demonstrate that measurements of antiviral adaptive immune responses may provide prognostic value and reflect the clinical course of polyomavirus infection.

Serologic surveys have examined MCV prevalence in various groups, including children (*16**,**17**,**23*), blood donors (*16**,**24*), MCC patients (*15**,**16**,**24*), and the general population (*15*), demonstrating that MCV infection is widespread among healthy adults. Further supporting widespread infection, Chen et al. recently reported evidence for high rates of MCV seroconversion among children 3–13 years of age (*23*). Significantly elevated anti-MCV capsid IgG levels are present in blood from MCC patients compared with healthy controls, suggesting the possibility of resurgent MCV replication among patients before MCC development (*15**,**16*). Antibodies against MCV large T and small T antigens are less sensitive for detecting exposure to this virus but may be useful in monitoring tumor progression among some virus-positive MCC patients (*12*).

To examine signs, symptoms, and diagnostic test results associated with primary MCV infection, we examined participants of the Pittsburgh Men’s Study, a component of the Multicenter AIDS Cohort Study (MACS). MACS recruited gay and bisexual men in 1984 and followed up at ≈6-month intervals with extensive symptom histories and physical examinations.

## Methods and Materials

### Study Population and Recruitment

For study population 1, stored samples from the Pittsburgh Men’s Study were examined. MACS was begun in 1984 to characterize the natural history of HIV infection in the United States. All study participants were homosexual and bisexual men >18 years of age who were followed up every 6 months with physical examinations and collection of serum, plasma, and peripheral blood mononuclear cells. To be eligible for our analysis, participants had to have remained in the Pittsburgh Men’s Study for at least 4 years. A subset of 564 study participants was selected for this study.

For study population 2, to search for a correlation between MCV and hepatitis B virus (HBV) infection, a set of 200 samples from participants of a community study of HBV infection in Qidong, People’s Republic of China, was examined. Persons in this study (73 male and 127 female) ranged in age from 12–35 years (median age 30 years). Roughly equal numbers of serum samples were selected for hepatitis B surface antigen (HBsAg) positivity and negativity; samples were matched for participant age and gender. Written informed consent was obtained, and all procedures and protocols were approved by the Institutional Review Board of the University of Pittsburgh.

### Seroconversion Analysis

Blood samples at study entry were examined for MCV IgG positivity. For those participants initially seronegative, a sample from the end of the study (≈4 years later) was tested, and, if MCV seropositive, intervening samples were tested to determine the time of seroconversion. MCV seroconversion was defined as the midpoint between last negative and first positive serum sample. For many of these seroconverters, additional follow-up samples were available in the years after the formal end of our 4-year cohort study. A corresponding study visit was randomly chosen from 82 MCV negative controls for comparison. Specifically, the distribution of visits for MCV seroconversion was used: 10 at visit 2 (V2), 1 each at V3, V4, V8, and V10; 4 at V5; 3 at V6; 8 at V7; and 2 at V9 (total 31). Using a 4:1 match at V2 and a 2:1 match for all other seroconversion visits, we selected82 controls, and their corresponding visit data were compared with the data at the first MCV-seropositive visit for the MCV seroconverters. Thus, MCV seroconverters and controls were temporally matched so that long-term events (e.g., AIDS progression) over the course of the cohort study would not bias the results.

### Virus-like Particle Production

Virus-like particles were produced in human embryonic kidney 293TT cells (*25*) (a kind gift of Chris Buck) as previously described (*26*). Viral protein (VP) 1 and VP2 genes were designed according to a silent codon modification scheme (GenBank accession nos. FJ548568–FJ54871) (*27*) and synthesized by Blue Heron Biotechnology (Bothell, WA, USA) based on MCV339 (accession no. EU375804) (*4*). BKV VLP produced in a baculovirus system (*28*) was a kind gift of John T. Schiller.

### ELISA

Serum or plasma samples were tested at 1:100 dilution in a blinded and randomized fashion for MCV VLP reactivity by using Immulon HB2 plates (Thermo Fisher Scientific, Waltham, MA, USA) coated overnight at 4°C with MCV virus-like particles at 100 ng of the protein per well in phosphate-buffered saline (PBS) and blocked with 0.5% nonfat dry milk for 2 hours at room temperature. Paired analysis of serum samples and plasma showed no significant differences on MCV ELISA of 100 μL of serum, diluted with PBS/0.5% milk, added to wells, and incubated at room temperature for 2 hours. Anti-MCV antibody was detected by using horseradish peroxidase–conjugated rabbit anti-human IgG or anti-human IgM (Dako, Glostrup, Denmark) diluted 1:6,000 with PBS/0.5% milk (100 μL incubated for 1 hour). TMB (3.3′,5.5′-tetramethylbenzidine) substrate (Sigma, St. Louis, MO, USA) was used to detect absorbance signal at 405 nm with reference wavelengths of 620 nm after 45 minutes of incubation. Assay optimization by using MCV virus-like particles for protein saturation curves were performed as previously described (*16*). Each determination was performed in duplicate, and OD values were adjusted by background subtraction by using wells without antigen as previously described (*29*). A detailed protocol for this assay is available from www.tumorvirology.pitt.edu/mcvtools.html.

### MCV ELISA Cutoff Determination

Cutoff values were based on results reported previously, which provides a detailed description (*16*). In brief, in this study, all samples with MCV IgG ELISA reactivity >0.2 OD units were found to have MCV-specific IgG specific for MCV virus-like particles but not heterologous polyomavirus virus-like particles by competition. Samples in the current study with MCV IgG OD values >0.2 units were considered positive without further testing. Previously, we also found human serum samples to have nonspecific reactivity up to 0.05 OD units that showed no specific competition. Thus, patient samples with MCV IgG ELISA <0.05 OD units in our current study were considered negative. Patient samples with MCV IgG ELISA results between 0.05 and 0.2 OD units were subjected to competition with MCV virus-like particles to determine if these titers were specific for MCV infection. If MCV reactivity for the sample was reduced by >50% by MCV virus-like particles competition, the sample was considered positive. Otherwise, the sample was considered negative for MCV antibodies.

### Virus-Like Particle Competition Assays

Competition experiments were performed by mixing soluble MCV or BK virus–like particles with diluted serum or plasma (100 ng per 100 μL) in 1.5-mL microcentrifuge tubes followed by incubation for 1 hour at room temperature. After incubation, serum samples were directly added to MCV VLP–coated plates, and ELISA was performed as described above.

### HBsAg and Core Antigen Antibody ELISA

Testing for HBsAg and hepatitis B core antigen antibody (HBc) were performed by using commercial ELISA kits (Abazyme, Needham, MA, USA, and Abnova, Taipei, Taiwan). Serum samples were diluted 1:100 with PBS and tested in duplicate. Results of the tests were interpreted by using cutoffs determined with negative and positive controls provided by manufacturers.

### Statistical Methods

All analyses were conducted by using GraphPad Prism software (GraphPad Software, Inc., La Jolla, CA, USA) and the Epi Info statistical calculator (wwwn.cdc.gov/epiinfo). Continuous data were analyzed by using a nonparametric Mann-Whitney test, and categorical data were analyzed by using a 2-sided Fisher exact test and by χ^2^ tests for trend.

## Results

### MCV Seroprevalence in MACS Participants

Study participants were 18–69 years old and were mostly white (96.2%). The mean age at baseline for participants was 32.8 years. Blood samples collected at entry were subjected to a battery of laboratory tests as described elsewhere (*30**,**31*). Patients were asked about symptoms during the preceding 6-month interval and evaluated for physical signs.

**Figure 1:**
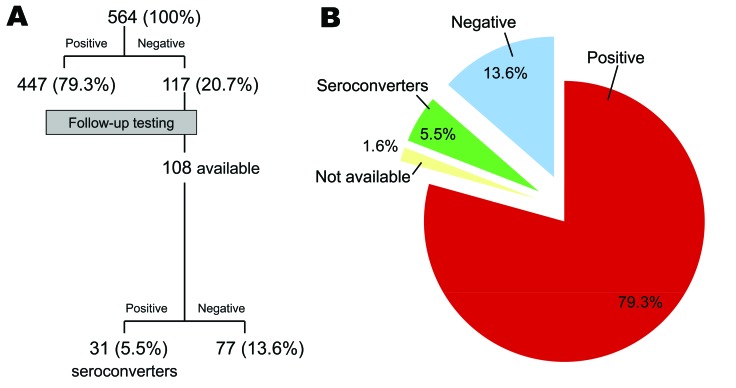
Merkel cell polyomavirus immunoglobulin G reactivity among Multicenter AIDS Cohort Study participants, Pittsburgh, Pennsylvania, USA. A) Flowchart of test results for 564 participants and for 108 participants with initial negative tests who were available for follow-up testing. B) Combined results for initial and follow-up testing.

**Figure 2:**
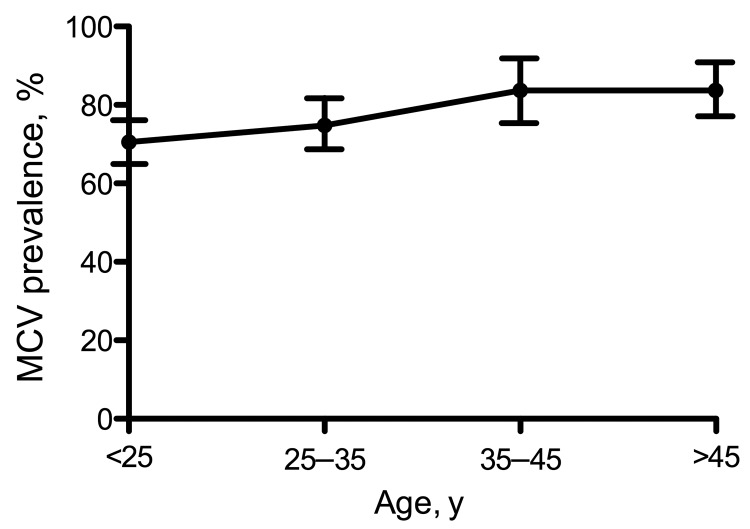
Age-dependent prevalence of Merkel cell polyomavirus antibodies among the Multicenter AIDS Cohort Study participants, Pittsburgh, Pennsylvania, USA. A small but significant linear trend for Merkel cell polyomavirus positivity with age among adult gay and bisexual men plateaued in the 35–45-year-old age group. Whiskers represent 95% confidence intervals.

Of 564 MACS participants, at enrollment 447 (79.3%) were positive for MCV IgG (Figure 1). The median MCV ELISA value for positive samples was 0.313 (range 0.013–2.451 OD units). The remaining 117 (20.7%) participants provided specimens that were MCV-antibody negative. A weak but significant increasing trend for MCV seropositivity with age (p = 0.036, χ^2^ test for trend) was found for young adults (25–35 years, n = 278) with a study entry prevalence of 74.8% that rose to 83.7% among men older than 45 years (n = 147) (Figure 2).

### Absence of Signs and Symptoms Associated with Prevalent MCV Infection

Comparing MCV-seropositive and seronegative men, we found no significant differences in HIV, hepatitis C virus, or syphilis status; blood count values; or other immunologic markers (Table 1). No correlation between MCV positivity and reported sexual activity was identified (data not shown). A significant association between MCV seropositivity and chronic hepatitis B virus infection was found at study entry: 31 (7.2%) of 447 MCV-seropositive participants were also positive for HBsAg as compared with 1 (0.8%) of 117 MCV-seronegative participants (odds ratio [OR] 8.1, 95% confidence interval [CI] 1.1–58.8). No significant association was found, however, between MCV and HBV exposure: 231 (51.7%) of 447 MCV-positive men, compared with 55 (47.0%) of 117 of MCV-negative men, were positive for HBc at study entry (OR 1.1, 95% CI 0.9–1.4). No other specific symptoms, signs, or blood test results were significantly associated with MCV positivity at entry, including rashes, diarrhea, fever, respiratory symptoms, or changes in total leukocyte count or cellular subpopulations (not shown).

**Table 1:** Routine blood test characteristics of MCV-infected and MCV-uninfected men at initial visit for Multicenter AIDS Cohort Study, Pittsburgh, Pennsylvania, USA*

Characteristic	MCV infected			MCV uninfected		p value
	No. tested	Value		No. tested	Value	
HIV positive, no. (%)	447	138 (31)		117	33 (28)	0.58
AIDS, subsequent, no. (%)	447	58 (13)		117	12 (10)	0.43
Hepatitis B surface antigen positive, no. (%)	446	31 (7)		117	1 (1)	0.01
Hepatitis B core antibody, no. (%)	446	231 (52)		117	55 (47)	0.36
RPR reactive, no. (%)	446	19 (4)		117	4 (3)	0.68
Leukocyte count, × 10^3^/mm^3^	443	6793.5		116	7111.2	0.22
Erythrocyte count, × 10^3^/mm^3^	443	5.0		116	5.0	0.53
Hemoglobin, g/dL	443	15.5		116	15.5	0.86
Hematocrit, %	443	45.8		116	46.0	0.45
Platelet count, × 10^3^/mm^3^	443	262.6		116	270.3	0.12
Neutrophils, %	443	59.1		116	59.5	0.38
Lymphocytes, %	443	35.6		116	35.5	0.41
Monocytes, %	300	4.0		69	3.7	0.33
Eosinophils, %	318	2.5		79	2.8	0.24
Cytomegalovirus Ab titer	447	239.9		117	232.1	0.86
Rubella Ab titer	447	92.0		117	87.6	0.16
IgA, mg/dL	447	360.6		117	361.4	0.44
IgG, mg/dL	447	2016.0		117	1945.9	0.91
IgM, mg/dL	447	220.3		117	227.6	0.62
CD4+, T-cells/μL	441	917.8		116	1004.2	0.09
CD8+, T-cells/μL	441	680.6		116	668.7	0.65

*MCV, Merkel cell polyomavirus; RPR, rapid plasma reagin; Ab, antibody; Ig, immunoglobulin.

### MCV Seroconversion Asymptomatic among Adult Men

To determine seroconversion, we tested 108 (92.3%) available plasma samples taken 4–5 years after study enrollment from the 117 initially MCV-seronegative persons. Of the 108 initially MCV-negative persons, 31 (5.5%) seroconverted (Figure 1), resulting in an incidence of 6.62/100 person-years.

MCV seroconversion at longitudinal follow-up visits was determined and infection was defined, for the purposes of this study, to occur at the midpoint between the last MCV-negative and the first MCV IgM- or IgG-positive blood sample. In general, MCV IgM peak levels preceded IgG seroconversion by 1 study visit or were concurrent with IgG seroconversion. Peak IgM levels (OD range 0.12–0.47), when present, were consistently lower than peak IgG levels (OD range 0.18–1.16). Once IgG seroconversion had occurred, none of the subsequent samples from these patients reverted to MCV IgG seronegativity.

**Figure 3:**
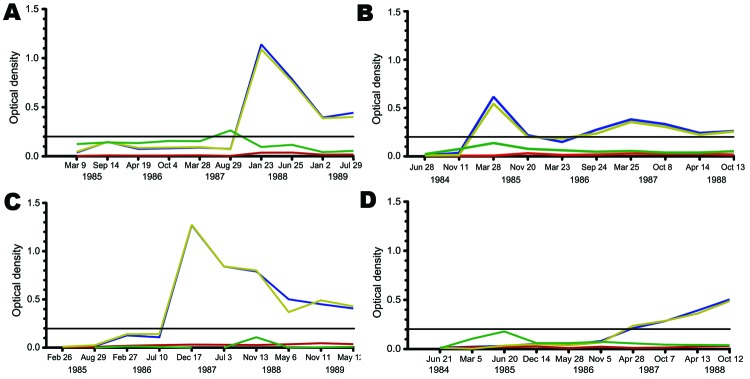
Representative patterns of Merkel cell polyomavirus (MCV) seroconversion among participants in the Multicenter AIDS Cohort Study, Pittsburgh, Pennsylvania, USA. Most participants showed MCV immunoglobulin (Ig) M (green line) and IgG (blue line) patterns similar to patient 1 (A) (MCV IgM peak immediately preceding IgG seroconversion) or patient 2 (B) (MCV IgM and IgG are concordant). For patient 3 (C), no IgM peak was detected during MCV IgG seroconversion. Delayed MCV IgG seroconversion, as seen with patient 4 (D), could also occur 1–2 years after an initial IgM spike. The black line represents the 0.2 optical density threshold value for MCV IgG positivity. The specificity of this test is shown by MCV virus-like particle (VLP) competition (red line) and BK virus (BKV) competition (gold line), in which MCV IgG titers are measured after plasma are preincubated with VLP antigen from the respective viruses. MCV IgG reactivity is markedly reduced by MCV competition but not BKV competition.

Typical patterns for seroconversion are shown in Figure 3. Patient 1 shows the most common pattern, in which IgM peaks immediately before a rise in IgG levels, while the next most common pattern, exemplified by patient 2, shows a concurrent IgM peak with IgG seroconversion. For 6 seroconverters, rises in IgG were not accompanied by a preceding increase in IgM reactivity (patient 3), possibly due to transient IgM peaks that resolved between the 2 blood collections. Finally, for 3 seroconverters, an MCV IgM peak was detected 1–2 years before the rise in MCV IgG titers, which suggests a prolonged period of infection before Ig class switching (patient 4).

To determine signs or symptoms associated with primary MCV infection, the date of seroconversion was identified for each of the seroconverters. Signs, symptoms, and laboratory values reported at the first MCV seropositive visit were then determined. Corresponding study visits (selected as described in Methods) from MCV seronegative controls were then examined. Symptoms, such as fever, rash, weight loss, fever, diarrhea, and cough present at the study visit were similar for MCV seroconverters and the control group (Table 2). Self-reported symptoms lasting >2 weeks during the 6 months before the MCV seroconversion visit were also not significantly different between patients and controls. Moreover, no statistically significant differences in erythrocyte, leukocyte, CD4+, CD8+ cell counts, or other clinical test values were identified between the 2 groups at the first MCV seropositive visit for patients or at comparable visits for controls (Table 3). Although not significant, a greater proportion of the MCV seroconverters were hepatitis B core antibody positive (63.3% vs. 44.4%) and had a marginally lower hemoglobin level (15.5 vs. 16.0 g/dL) as compared with controls. No DNA of the virus was detected by real-time PCR in the plasma for 4 participants at the time of seroconversion (data not shown).

**Table 2:** Signs and symptoms at seroconversion study visit or during previous 6 months for MCV seroconverters and controls matched by study visit among participants in the Multicenter AIDS Cohort Study, Pittsburgh, Pennsylvania, USA*

Signs or symptom	Seroconverters			Controls		p value
	No. evaluated	No. (%) with sign or symptom		No. evaluated	No. (%) with sign or symptom	
Shortness of breath >2 weeks	31	1 (3.2)		75	8 (10.7)	0.28
Shortness of breath at time of visit	28	1 (3.6)		73	6 (8.2)	0.67
Cough >2 weeks	31	1 (3.2)		75	4 (5.3)	1
Cough now	31	0		75	3 (4.0)	0.55
Sore throat >2 weeks	31	1 (3.2)		75	5 (6.7)	0.67
Sore throat now	31	1 (3.2)		75	2 (2.7)	0.50
Rash >2 weeks	31	2 (6.5)		75	4 (5.3)	1
Rash now	31	3 (9.7)		75	4 (5.3)	0.41
Bruising >2 weeks	31	0		75	4 (5.3)	0.32
Bruising now	31	0		75	3 (4.0)	0.55
Fatigue >2 weeks	31	0		75	5 (6.7)	0.32
Fatigue now	31	1 (3.2)		75	4 (5.3)	1
Weight loss >10 pounds	31	0		75	1 (1. 3)	1
Weight loss now	31	0		75	0	NA
Diarrhea >2 weeks	31	2 (6.5)		75	0	0.08
Diarrhea now	31	1 (3.2)		75	0	0.29
Fever >2 weeks	31	0		75	0	N/A
Fever now	31	0		75	0	N/A
Lymphadenopathy >2 weeks	31	3 (9.7)		75	5 (6.7)	0.69
Lymphadenopathy now	31	3 (9.7)		75	5 (6.7)	0.69
Night sweats >2 weeks	31	0		75	4 (5.3)	0.32
Night sweats now	31	0		75	2 (2.7)	1.0
Headache >2 weeks	31	1 (3.2)		75	3 (4.0)	1.0
Headache now	31	1 (3.2)		75	2 (2.7)	1.0

*MCV, Merkel cell polyomavirus; NA, not applicable.

**Table 3:** Routine blood test characteristics for MCV seroconverters and controls matched by study visit among participants in the Multicenter AIDS Cohort Study, Pittsburgh, Pennsylvania, USA*

Characteristic	MCV seroconverters			Controls		p value
	No. tested	Value		No. tested	Value	
HIV positive, no. (%)	31	12 (38.7)		82	19 (23.2)	0.10
AIDS, subsequent, no. (%)	31	5 (16.1)		82	7 (8.5)	0.31
Hepatitis B surface antigen positive, no. (%)	30	0 (0)		81	1 (1.2)	1.00
Hepatitis B Core antigen antibody positive, no. (%)	30	19 (63.3)		81	36 (44.4)	0.08
RPR reactive, no. (%)	31	1 (3.2)		73	1 (1.4)	0.50
Leukocytes count, × 10^3^/mm^3^	31	7174.2		75	6978.7	0.26
Erythrocytes count, × 10^3^/mm^3^	31	5.1		75	5.2	0.13
Hemoglobin, g/dL	31	15.5		75	16.0	0.07
Hematocrit, %	31	46.7		75	47.7	0.15
Platelet count, × 10^3^/mm^3^	31	247.3		75	259.6	0.14
Polymorphonuclear lymphocytes, %	31	58.7		75	58.1	0.61
Lymphocytes, %	31	32.6		75	32.9	0.83
Monocytes, %	29	5.9		72	5.5	0.22
Eosinophils, %	30	2.6		74	3.1	0.39
Atypical lymphocytes, %	29	1.2		71	1.1	0.56
Cytomegalovirus Ab titer	12	168.5		44	306.4	0.34
Rubella Ab titer	12	73.6		45	75.4	0.72
IgA, mg/dL	12	308.2		45	295.1	0.70
IgG, mg/dL	12	1,117.2		45	1151.5	0.93
IgM, mg/dL	12	178.8		45	172.5	0.75
CD4+, T-cells/μL	26	925.1		65	900.1	0.85
CD8+, T-cells/μL	26	637.7		65	564.5	0.22

*MCV, Merkel cell polyomavirus; RPR, rapid plasma reagin; Ab, antibody; Ig, immunoglobulin.

### Longevity of MCV IgG Responses

**Figure 4:**
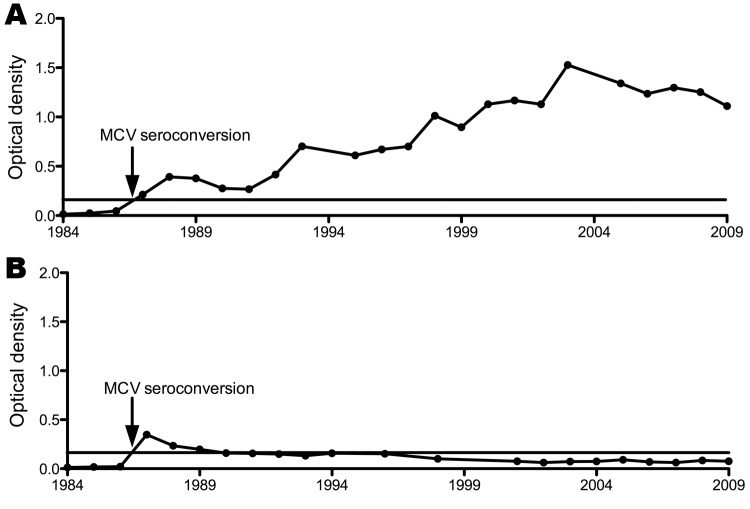
Two general patterns for Merkel cell polyomavirus (McV) immunoglobulin G levels after seroconversion among participants in the Multicenter AIDS Cohort Study, Pittsburgh, Pennsylvania, USA: a gradual increase over the 25-year period (A, patient 5) or a multiyear decline during 25-year follow up (B, patient 6). Horizontal line represents the 0.2 optical density threshold value for positivity.

Plasma from 17 of the 31 MCV seroconverters with known dates of infection was available for long-term follow-up (ranging from 7–25 years). Two general patterns of anti-MCV antibody reactivity were seen: 11 (64.7%) patients demonstrated robust MCV seroconversion, with slowly increasing levels of MCV IgG over time (Figure 4). The second pattern (6 patients) revealed a transient increase in MCV IgG >0.2 OD units at the time of seroconversion, which declined over 1–2 years and generally remained <0.2 OD units. Despite this decline, all 6 retained readily detected MCV IgG as measured by MCV VLP competition assays.

### Lack of Correlation between Hepatitis B Virus Infection and MCV Status

Prevalent MCV seropositivity at study entry was only significantly associated with a positive test result for chronic HBsAg carriage. To examine the possible role of MCV infection in influencing chronic HBV infection, we tested 93 HBsAg-positive and 107 negative serum samples collected in a convenience sample from Qidong residents. Among these samples the prevalence of MCV antibody positivity was 66% with mean OD 0.452 (range 0.01–2.19). Rates of MCV positivity for HBsAg positive and negative samples were 61% and 70%, which were not statistically different (Table 4). Among the 107 HBsAg negative participants, MCV prevalence was not statistically significantly different for those exposed (HBc negative, n = 9) and not exposed (HBc negative, n = 84) to HBV infection (75% vs. 71%, respectively).

**Table 4:** MCV prevalence among 200 Qidong, China, residents with and without hepatitis B virus surface antigen and hepatitis B virus core antibodies*

Characteristic†	No. residents	MCV positive, no. (%)	MCV negative, no. (%)
HBsAg positive/B core antibody positive	84	51 (61)	33 (39)
HBsAg positive/B core antibody negative	9	5 (56)	4 (44)
HBsAg negative/B core antibody positive	8	6 (75)	2 (25)
HBsAg negative/B core antibody negative	99	70 (71)	29 (29)

*MCV, Merkel cell polyomavirus, HBsAg, hepatitis B virus surface antigen. †NS for comparison of either HBsAg or hepatitis B core antibody with MCV positivity.

## Discussion

Our study makes use of the MACS cohort study to evaluate MCV seroprevalence and seroconversion among adult men at risk for HIV infection. While MCC occurrence is elevated among HIV-positive persons (*32*), it is still an uncommon cancer and no cases of MCC were reported among the participants selected from MACS for this study. We found no correlation between MCV infection and HIV status or AIDS in our study. MCV seroconversion wa*s* not associated with signs or symptoms of illness in adult gay and bisexual men. MCV prevalence plateaued for men 35–45 years of age in our study, which is consistent with primary MCV infection occurring mainly among children and young adults (*23*). We found MCV prevalence among participants was 79.3% with a 6.6% annual seroconversion rate, which suggested widespread circulation of the virus. Our study suggests that MCV infection is a highly prevalent infection among adults that is often asymptomatic. We cannot exclude rare illnesses occurring from primary MCV infection, however, or illness mild enough not to be reported by our cohort participants. These results, and those of others, indicate that active MCV transmission is common even though MCV-related cancer is rare (*33*).

Signs and symptoms for primary MCV infection were not found in our study. An important caveat is that MACS participants self-reported symptoms at ≈6-month intervals, and minor symptoms may have been forgotten between study visits. MACS is a closely monitored cohort study designed to study risk factors and natural history of HIV in homosexual and bisexual men in the United States. Participants in this study were all sexually active adult men, most of whom were already positive for MCV, and so caution is needed in generalizing our results to other populations (e.g., women, children, non-US populations). We cannot exclude, for example, the possibility of symptoms or disease after primary pediatric MCV infection. Weak correlations that did not reach a level of significance in our study, such as lower hemoglobin and hematocrit values after MCV seroconversion, might be reconciled by testing in other cohorts.

We did find an unexpected correlation between prevalent MCV infection and chronic HBV carriage for MACS participants. HBc positivity, however, was not elevated. When MCV seroconverters were examined, no correlation was found between MCV infection and HBsAg positivity, and only a weak but nonsignificant association was present for HBc values. It is likely that most of the MACS men were exposed to HBV as adults through unprotected sex or parenteral exposure. None of our other comparisons suggest that either of these routes of infection is significant for MCV, although we can infer that MCV infection (a childhood infection that primarily occurs before onset of sexual activity) (*23*) likely preceded HBV infection in most participants.

To further investigate the relationship between MCV and HBV infection, we examined HBV-hyperendemic samples from eastern China that likely represent mainly vertical or early childhood horizontal HBV infections. No correlation with MCV infection was found. Because of selection to ensure sufficient numbers of HBV-exposed participants, the Qidong study group cannot be assumed to represent a community serosurvey. Nonetheless, our results indicate widespread MCV infection among Asian adults similar to that seen for North Americans. It is unlikely that MCV and HBV are biologically linked in any significant manner, but caution is needed in interpreting these results since modes of HBV infection for MACS and Qidong participants are different.

MCV appears to be a life-long, chronic infection that may cause continuous antigen stimulation. Recent studies have shown that detection of MCV antibodies is improved by use of conformational epitopes present in VLP ELISA (*16**,**23**,**34*). Detection of MCV IgG by virus-like particles ELISA is persistent for up to 25 years after seroconversion, making it unlikely that seronegative participants were exposed to MCV and subsequently lost detectable antibodies. While only a portion of skin samples from healthy persons have viral DNA detectable by PCR (*35**,**36*), more sensitive techniques show persistent viral DNA in skin samples over a time scale of months and possibly years (*19*).

Our study indicates that MCV is one of a burgeoning number of newly recognized viruses that are part of the normal human flora (*19**,**37**–**39*). MCV infection among adults is generally silent and not associated with common signs, symptoms, or laboratory measures for infection. This virus, nonetheless, directly contributes to one of the most deadly human skin cancers, which illustrates that common commensal viral infections can contribute to the etiology of chronic diseases under unusual circumstances, such as virus mutation together with loss of immune surveillance.
